# Supplementary material for: Amyloid Aβ25-35 Aggregates Say ‘NO’ to Long-Term Potentiation in the Hippocampus through Activation of Stress-Induced Phosphatase 1 and Mitochondrial Na+/Ca2+ Exchanger
Source: Int J Mol Sci. 2022 Oct 6;23(19):11848. doi: 10.3390/ijms231911848 (PMC9570122; doi:10.3390/ijms231911848)
Supplement: Supplementary file 1 [file ijms-23-11848-s001.zip › ijms-1917196-supplementary.pdf]

Supplemental Data

Amyloid Aβ$_{25-35}$ aggregates say 'NO' to long-term potentiation in the hippocampus through activation of stress-induced phosphatase 1 and mitochondrial Na^+/Ca^{2+} exchanger

Alexander V. Maltsev [1*], Anna B. Nikiforova [2], Natalia V. Bal [1], Pavel M. Balaban [1]

[1] – Institute of Higher Nervous Activity and Neurophysiology, Russian Academy of Sciences, 117485, Butlerova 5A, Moscow, Russia.
[2] – Institute of Theoretical and Experimental Biophysics, Russian Academy of Sciences, 142290, Pushchino, Moscow region, Russia.
* - correspondence address: alex.maltsev2504@gmail.com

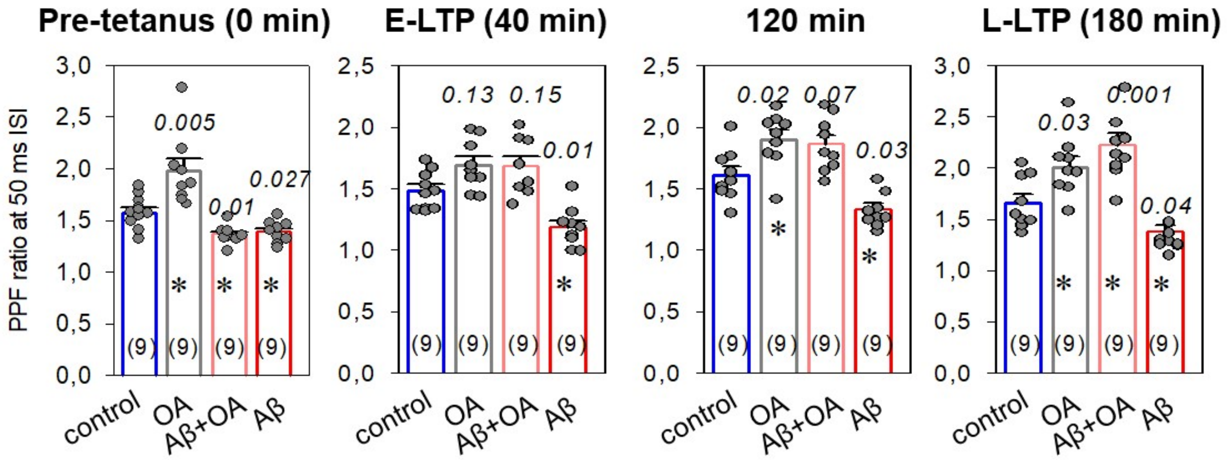

Suppl. Fig. 1. Kinetics of paired-pulse facilitation (PPF) changes in okadaic acid (100 nM) (OA)-pretreated slices at different time point (for pre-tetanic levels, 0 min; 40 min after LTP induction (E-LTP), 120 min after LTP induction, and at the L-LTP, 180 after tetanic stimulation. * denotes $p < 0.05$.

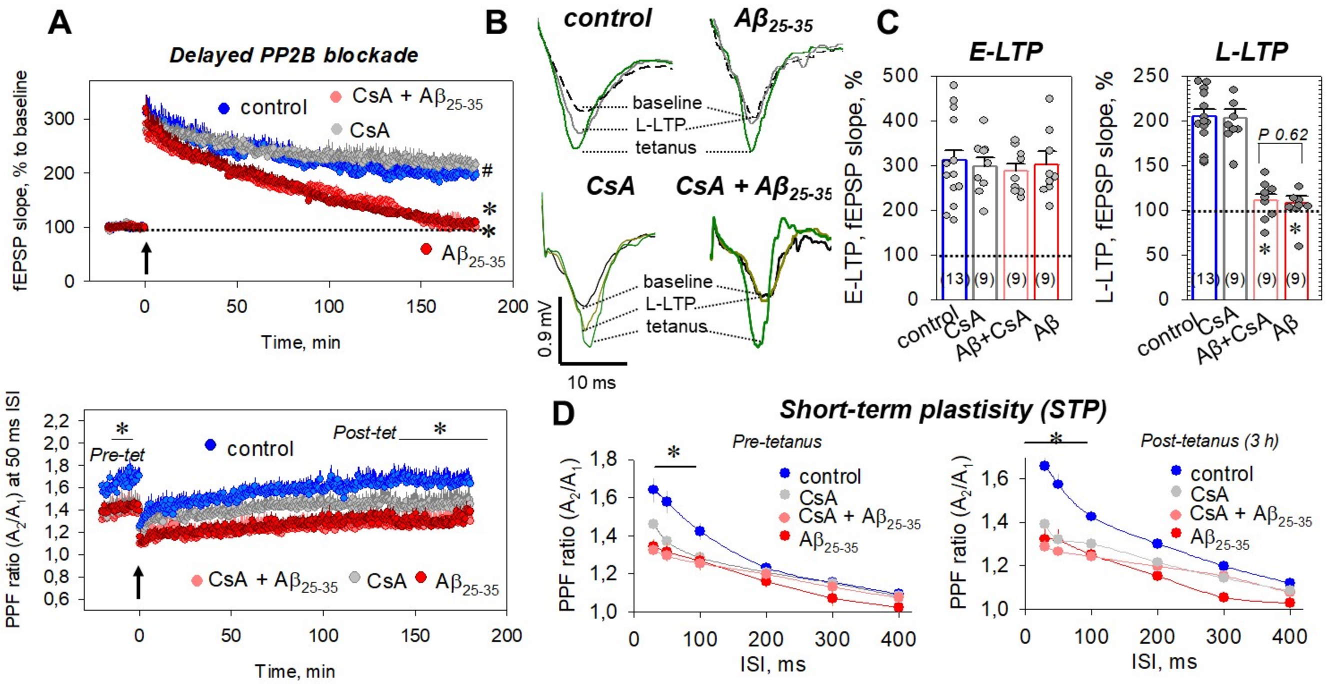

Suppl. Fig. 2. The delayed PP2B blockade during the Aβ25-35-dependent impairment of hippocampal synaptic plasticity. (A) – averaged curves for control slices (blue circles, n = 13), cyclosporin A (CsA)-treated slices (5 µM, gray circles, n = 9), Aβ25-35-treated slices (dark red circles, n = 9) and CsA+ Aβ25-35-treated slices (red circles, n = 9). Arrow at 0 min indicates start of tetanic stimulation, dotted line 100% is normalized fEPSPs corresponding to the pre-tetanic level. Bottom panel represents paired-pulse facilitation (PPF) curves at the 50 ms ISI corresponding to the curves from top panel. (B) – typical fEPSP responses of studied groups for pre-tetanic levels (baseline), at the start of post-tetanic recordings (tetanus) and 180 min after the LTP induction (L-LTP). (C) – Summarized statistics for (A) curves during early phase (E-LTP, 0-3 min) and late phase (L-LTP, 178-180 min) of long-term potentiation. * denotes p<0.05 for comparing of the group with control, # indicates p<0.05 for comparison of groups between themselves. The number of independent experiments is indicated in parentheses. (D) – PPF ratios for different interstimulus intervals (30 ms – 400 ms) before tetanic stimulation (left panel, pre-tetanus) and for L-LTP, 3h after tetanus (right panel, post-tetanus).

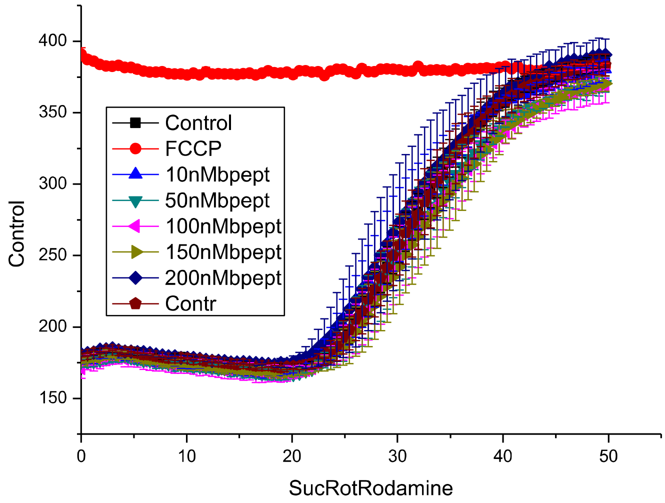

Suppl. Fig. 3. Curves of detection of mitochondrial transmembrane potential $\Delta\Psi_m$ using rhodamine 123 fluorescence in the RBM suspension (substrates: succinate 5 mM + rotenone 1 µM) for three independent measurements. FCCP (200 nM), an established oxidative phosphorylation uncoupler, was used as negative control for disruption of $\Delta\Psi_m$, bpept – Aβ25-35 in different concentrations (10 – 200 nM). Axis Y – fluorescence, a.u., axis X – time, min.

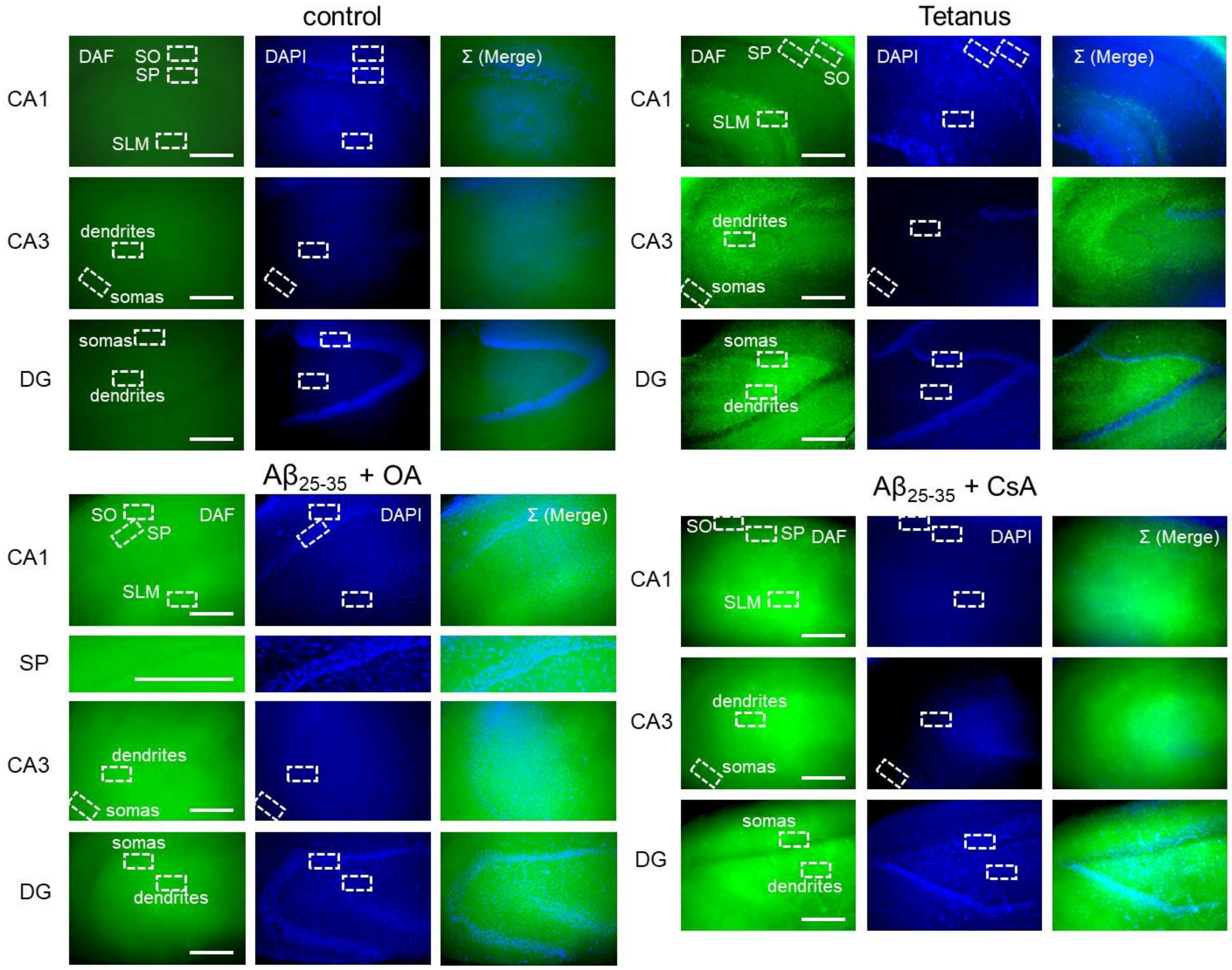

Suppl. Fig. 4. NO staining by DAF-FM dye in the control, tetanized slices, as well OA (100 nM)+A$\beta_{25\text{-}35}$- and CsA (5 µM)+A$\beta_{25\text{-}35}$-treated slices. SP – Stratum pyramidale, SLM – Stratum lacunosum-moleculare (SLM), DG – dentate gyrus. Blue fluorescence channel indicates staining of cell nuclei by DAPI. Scale bars is 300 µm.

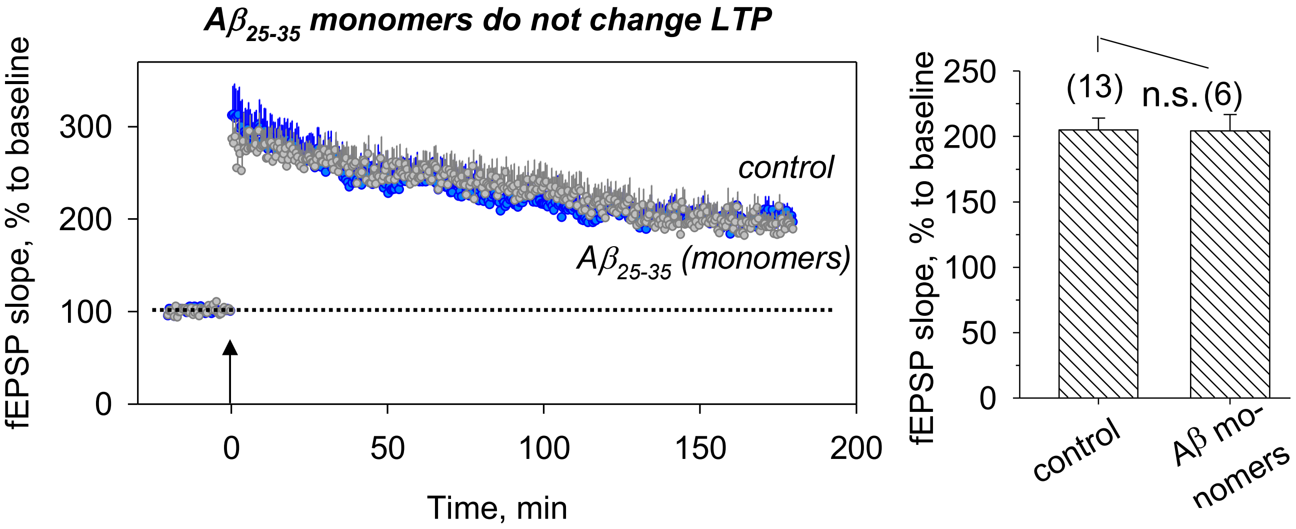

Suppl. Fig. 5. The 1h incubation of hippocampal slices in the freshly prepared A$\beta_{25\text{-}35}$ solution (A$\beta_{25\text{-}35}$ monomers, 50 nM) had no influences on the LTP kinetics.

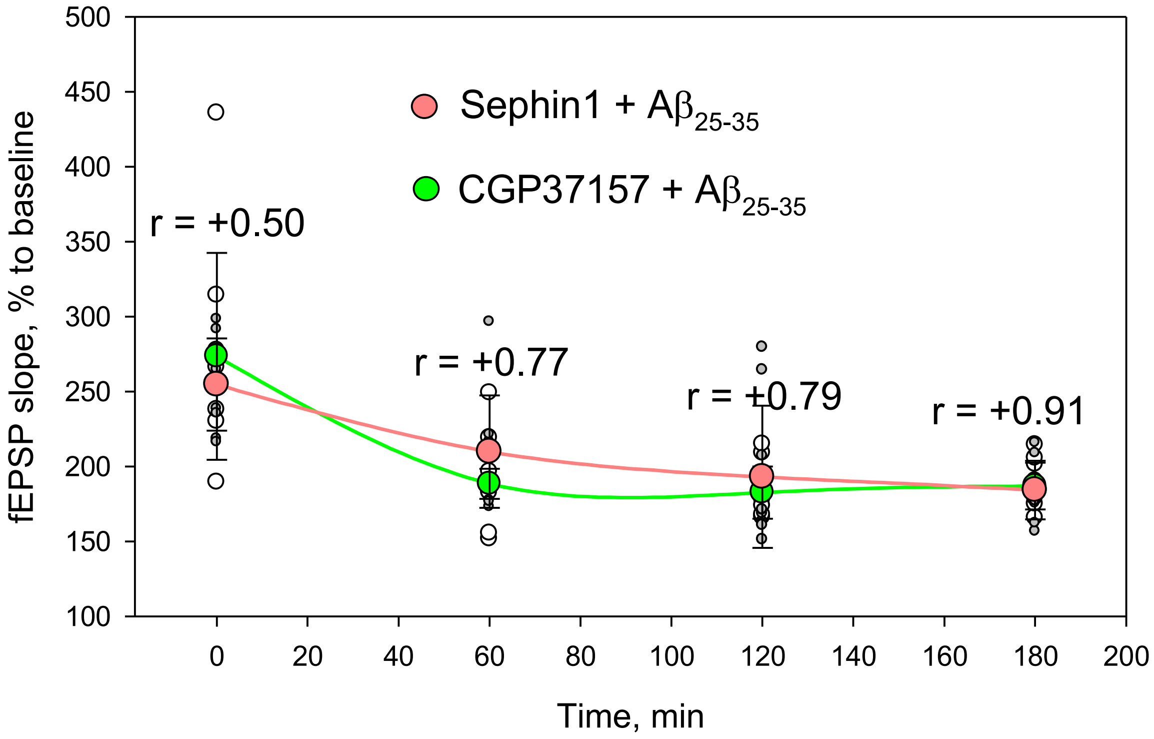

Suppl. Fig. 6. Pearson's correlation coefficients for LTP kinetics after tetanic stimulation (0-180 min) of hippocampal slices co-incubated in the presence of 50 nM $A\beta_{25-35}$ aggregates and 10 µM sephin1 (red circles and red curve timeline, gray circles – individual points, n = 9), or 10 µM CGP37157 (green circles and green curve, white circles – individual points, n = 9). The positive Pearson's coefficients in respect to the LTP kinetics reflect that there is a positive correlation between PP1α and mNCX blockade and the prevention of $A\beta_{25-35}$ aggregate-dependent LTP suppression.
